# Feasibility and Outcomes of Upgrading to Left Bundle Branch Pacing in Patients With Pacing-Induced Cardiomyopathy and Infranodal Atrioventricular Block

**DOI:** 10.3389/fcvm.2021.674452

**Published:** 2021-06-14

**Authors:** Yang Ye, Shengjie Wu, Lan Su, Xia Sheng, Jiefang Zhang, Bei Wang, Parikshit S. Sharma, Kenneth A. Ellenbogen, Yangang Su, Xueying Chen, Guosheng Fu, Weijian Huang

**Affiliations:** ^1^Department of Cardiology, Sir Run Run Shaw Hospital, College of Medicine, Zhejiang University, Hangzhou, China; ^2^Key Laboratory of Cardiovascular Intervention and Regenerative Medicine of Zhejiang Province, Hangzhou, China; ^3^Department of Cardiology, The First Affiliated Hospital of Wenzhou Medical University, Wenzhou, China; ^4^Key Lab of Cardiovascular Disease of Wenzhou, Wenzhou, China; ^5^Department of Cardiac Echocardiology, Sir Run Run Shaw Hospital, College of Medicine, Zhejiang University, Hangzhou, China; ^6^From the Division of Cardiology, Rush University Medical Center, Chicago, IL, United States; ^7^Virginia Commonwealth University Medical Center, Richmond, VA, United States; ^8^Department of Cardiology, Shanghai Institution of Cardiovascular Disease, Zhongshan Hospital, Fudan University, Shanghai, China

**Keywords:** cardiac pacing, atrioventricular block (AVB), pacing-induced cardiomyopathy (PICM), heart failure (HF), His bundle pacing (HBP), left bundle branch pacing (LBBP), cardiac resynchronization therapy (CRT)

## Abstract

His bundle pacing (HBP) can reverse left ventricular (LV) remodeling in patients with right ventricular (RV) pacing-induced cardimyopathy (PICM) but may be unable to correct infranodal atrioventricular block (AVB). Left bundle branch pacing (LBBP) results in rapid LV activation and may be able to reliably pace beyond the site of AVB. Our study was conducted to assess the feasibility, safety, and outcomes of permanent LBBP in infranodal AVB and PICM patients. Patients with infranodal AVB and PICM who underwent LBBP for cardiac resynchronization therapy (CRT) were included. Clinical evaluation and echocardiographic and electrocardiographic assessments were recorded at baseline and follow-up. Permanent LBBP upgrade was successful in 19 of 20 patients with a median follow-up duration of 12 months. QRS duration (QRSd) increased from 139.3 ± 28.0 ms at baseline to 176.2 ± 21.4 ms (*P* < 0.001) with right ventricular pacing (RVP) and was shortened to 120.9 ± 15.2 ms after LBBP (*P* < 0.001). The mean LBBP threshold was 0.7 ± 0.3 V at 0.4 ms at implant and remained stable during follow-up. The left ventricular ejection fraction (LVEF) increased from 36.3% ± 6.5% to 51.9% ± 13.0% (*P* < 0.001) with left ventricular end-systolic volume (LVESV) reduced from 180.1 ± 43.5 to 136.8 ± 36.7 ml (*P* < 0.001) during last follow-up. LBBP paced beyond the site of block, which results in a low pacing threshold with a high success rate in infranodal AVB patients. LBBP improved LV function with stable parameters over the 12 months, making it a reasonable alternative to cardiac resynchronization pacing via a coronary sinus lead in infranodal AVB and PICM patients.

## Introduction

Right ventricular pacing (RVP) leads to left ventricular (LV) dyssynchrony and may result in symptoms of heart failure (HF), a syndrome known as pacing-induced cardiomyopathy (PICM) (1, 2). PICM is an important and under-recognized cause of cardiomyopathy and may occur in up to 5–20% of patients with chronic RVP (2, 3). Cardiac resynchronization therapy (CRT) utilizing biventricular pacing (BVP) is recommended for patients who develop a PICM and can result in an improvement in cardiac function and LV remodeling (4). However, the clinical improvement after upgrade from RVP to BVP is limited by cardiac venous anatomy and LV lead positioning (5). BVP may not be the best strategy to maintain synchrony in patients with a native narrow QRS and may not overcome the challenges associated with non-physiological ventricular activation in patients with a narrow QRS and atrioventricular block (AVB) (6). As the most physiological ventricular pacing strategy, permanent His bundle pacing (HBP) improves LV function in PICM patients (7, 8), however is limited by variable success rate, potential high His bundle capture thresholds, low R-wave amplitudes, atrial oversensing, as well as an increased risk of lead revision with late threshold rise (9–11). The HBP has a lower success rate among patients especially with infranodal AVB due to pacing proximal to the site of block and the possibility of the threshold rise due to the progression of conduction disease (9). Left bundle branch pacing (LBBP) was first described in 2017 (12) and has demonstrated clinically promising results including the safety, efficacy, and outcomes in various patient populations with low and stable thresholds (13, 14). LBBP preserves rapid LV activation, and a recent case report demonstrated that LBBP resulted in reverse LV remodeling in a patient with PICM and infranodal AVB (15). LBBP also achieved electric resynchronization in HF and left branch bundle block (LBBB) patients with low and stable pacing thresholds (16). Given the location of LBBP and the theoretical advantage of pacing distal to the site of conduction block, this could be well-suited for patients with infranodal AVB.

The objective of our multicenter study was to assess the feasibility, safety, and clinical outcomes of LBBP in patients with infranodal AVB undergoing a device upgrade for PICM as a result of chronic RVP.

## Methods

### Patient Selection

This was a retrospective multicenter study including all consecutive patients undergoing LBBP pacing between December 2017 and June 2019 at three centers (Wenzhou, Hangzhou, and Shanghai) meeting the inclusion criteria. Patients >18 years of age who met the following inclusion criteria were enrolled in this study: (1) PICM patients, which was defined as a >10% decrease in left ventricular ejection fraction (LVEF) after chronic RVP resulting in LVEF ≤ 50%. (2) The pacing percentage of RVP was >40%. (3) All patients were assessed for the site of conduction block. Then, the patients in whom infranodal AVB was confirmed, LBBP was attempted in order to pace beyond the site of block. Patients with other causes of LV dysfunction, including myocardial infarction, valvular heart disease (>15%), frequent ventricular premature depolarizations, and uncontrolled hypertension (>160/100 mmHg), were not defined to have PICM and were excluded (2). All patients had received standard medical treatments for HF at least 3 months before the upgrade. The hospital institutional review board approved the study procedure, and all patients were provided informed consent and demonstrated the understanding of LBBP therapy as a non-standard approach to achieve physiological pacing. Data analysis was approved by the institutional review board at all three institutions and was retrospectively analyzed.

### Implantation Technique and Procedure Details

During the LBBP implantation, intracardiac electrograms (EGM) along with a 12-lead surface ECG GE CardioLab EP Recording System 2000; GE, Milwaukee, Wisconsin, USA. LabSystem PRO, Bard Electrophysiology, 196 Lowell, MA, USA were recorded. The techniques for HBP and LBBP were described in detail in our prior study (17, 18). Briefly, the 3830 lead (SelectSecure, Medtronic, Minneapolis, MN, USA) was advanced through the C315HIS delivery sheath to a spot for unipolar His bundle (HB) site mapping and pacing. Then, the 3830 lead was further advanced to a spot on the interventricular septum that is 1–1.5 cm apical along an axial line between the distal HB site and right ventricular (RV) apex in the right anterior oblique projection on fluoroscopy. The lead was then advanced deep into the septum in order to achieve left conduction system capture. LBBP was confirmed (19) and differentiated from left ventricular septal pacing (LVSP) by the criteria published previously (20).

Infranodal AVB is defined as the intra-Hisian and infra-Hisian block shown as a split His, His potential to ventricle interval (HV) prolongation, or HV dissociation. In patients without an underlying escape rhythm, the pacing rate was decreased to 30–35 bpm to assess for an escape rhythm. If patients had a ventricular escape rhythm due to sinus bradycardia, atrial pacing was used to help test intrinsic conduction to determine the site of block. A HBP lead was used to record the His potential to help assess the site of block.

In patients with AVB, the left bundle branch (LBB) potential was recorded (21), proceeding the ventricular electrogram and pacing at a rate of 130 beats/min (0.5 V above LBB capture threshold) to test the refractory period of the distal conduction system and ensuring capture and 1:1 conduction. In those with LVEF <35%, a decision to implant an implantable cardiac defibrillator (ICD) was based on shared decision making between the implanter and the patient. LBB potential (s) and/or ventricular electrograms were assessed and lead parameters were analyzed. The pacing lead was then connected with a device (described in Supplementary Table 1). At the physician's discretion, the device was programmed to LBBP only.

### ECG Evaluation

Electrical dyssynchrony was assessed by QRS width and axis. They were compared between native, RVP, and LBBP configurations. QRS duration (QRSd) was measured in 12 contemporary ECG leads. Paced QRSd was measured from the pacing stimulus to the end of QRS complex. Normal frontal QRS axis was defined as −30° to 90°, left axis deviation (LAD) as −90° to −30°, moderate right axis deviation (RAD) as 90°-180°, and superior RAD as 180°-270°. The precordial lead transition in which the precordial lead R-wave amplitude is equal to or greater than the S wave amplitude was recorded.

### Clinical Assessment and Follow-Up

Patients underwent regular follow-up at 3 and 6 months and annually post implantation. Functional status was assessed by the New York Heart Association (NYHA) classification. Device thresholds were checked and adjusted as needed to maximize battery longevity. Echocardiograms were performed as clinically indicated for follow-up. At follow-up visits, R-wave amplitude, capture threshold, pacing impedance, and the percentage of LBBP were collected. Standard echocardiographic indices including LVEF, left ventricular end-diastolic diameter (LVEDD), left ventricular end-systolic volume (LVESV), and valvular regurgitation were acquired by an experienced physician in accordance with the American Society of Echocardiography guidelines (7). Lead-related complications such as a significant increase in pacing threshold, lead dislodgment, or loss of capture were routinely tracked. Heart failure hospitalization (HFH) was determined by the following criteria: admission to hospital for >24 h due to worsening symptoms of HF and requiring intravenous diuretics or intravenous inotropic medications. The echocardiographic response was defined as ≥5% increase in LVEF. Super response was defined as an absolute improvement in LVEF by ≥20% or improvement of LVEF to 50% from a baseline value of <35% (17).

### Statistical Analysis

Continuous variables were expressed as mean ± SD or median [interquartile range (IQR)]. Paired comparisons were made with a Student's *t*-test if the data were normally distributed and with the Wilcoxon signed-rank test for non-parametric data. Paired categorical data (NYHA functional class) were compared with the Wilcoxon test. For echocardiographic LVEF, LVEDD, LVESV, and parameters (threshold, sensed R-wave amplitude, and the percentage of LBBP) that were collected at baseline and later multiple different time points, univariate analysis of variance for repeated measures was used to assess the effects of LBBP. A *P* value ≤ 0.05 was considered statistically significant. Data analyses were performed using SPSS version 20.0 (SPSS, Chicago, IL, USA).

## Results

### Patient Characteristics and Implantation Results

**Figure 1:**
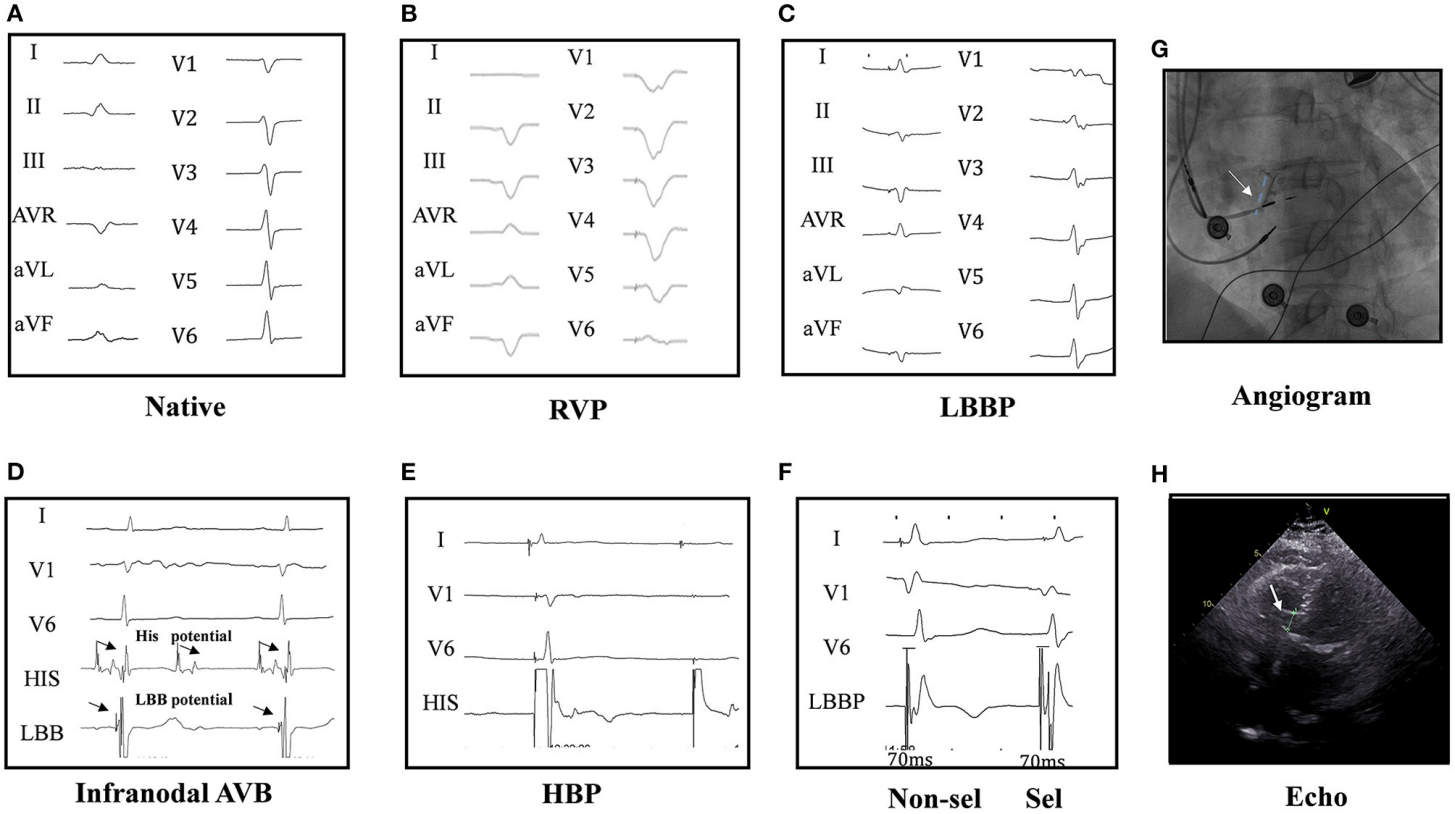
Twelve-lead ECG and intracardiac electrogram (EGM) in a patient. The native cardiac rhythm **(A)**; twelve-lead ECG by right ventricular pacing (RVP) **(B)**; the ECG by left bundle branch pacing (LBBP) **(C)**; narrow ECG QRS with LBB potential in intracardiac electrograms (EGM at the bottom) in a infranodal atrioventricular block (AVB) patient **(D)**. His bundle pacing (HBP) could not pace beyond the block site (2.5 V at 0.4 ms) **(E)**. Selective (0.6 V at 0.4 ms) and non-selective (0.5 V at 0.4 ms) LBBP **(F)** and the angiogram of the sheath to assess the depth of the LBB LBBP lead **(G)** are shown. Echocardiography showed the lead was fixed in the left ventricular septum **(H)**, and LBBP but not HBP could pace beyond the block site in one patient of AVB with infranodal block. HBP at 2.5 V/0.4 ms could induce the loss of capture (2:1) **(E)**, while LBBP could induce pacing (1:1) **(F)**, indicating LBBP but not HBP could pace beyond the block site in infranodal AVB patients. His and LBB potential were seen in the pacing location **(D)**.

Twenty PICM patients with confirmed infranodal AVB were referred for LBBP upgrade. One patient had failed LBBP lead fixation and was left with LVSP (22). Thus, 19 infranodal AVB patients with successful LBBP upgrade were included. As noted in Figure 1, in a patient with infranodal AVB, LBBP but not HBP resulted in conduction system capture beyond the site of block. The baseline characteristics of the study population are demonstrated in Table 1. The mean age of the successfully implanted patients was 70.2 ± 8.6 years with 57.9% male. The indication for permanent RVP was mostly complete AVB. The median percentage of RVP was 100% (IQR: 97% to 100%), and the mean duration of RVP was 76.4 ± 33.5 months before upgrade to LBBP. Of 19 patients successfully implanted, 52.6% (10/19) patients had LBB potentials with the mean potential to ventricle interval of 19.7 ± 6.6 ms. The median fluoroscopy duration for LBBP lead implantation in all these 19 patients was 7 min (IQR: 6 to 7 min), and the median total procedural time for implantation was 80 min (IQR: 70 to 100 min). The median follow-up after upgrade to LBBP was 12 months (IQR: 12−12 months). The percentage of LBBP was 97.0% ± 16.9% during the median 12-month follow-up. The mean threshold for LBBP capture was 0.7 ± 0.3 V at 0.4 ms and the R-wave amplitude was 12.7 ± 4.2 mv. The mean lead impedance at implant was 625.1 ± 118.8 Ω. There were no complications during the procedure.

**Table 1:** Baseline clinical characteristics of patients.

	***N* (%)/mean ± SD/** **median (IQR)**
Successful LBBP	19 (95.0)
Age (years)	70.2 ± 8.6
Male (%)	11 (57.9)
Coronary artery disease	1 (5.3)
Atrial fibrillation	2 (10.5)
Hypertension	6 (31.6)
LVEF (pre-RVP, %)	62.0 ± 6.6
Duration of RVP (months)	75.5 ± 33.3
Percentage of RVP %	100 (97–100)
AVB	
Complete AVB	14 (73.7)
Second degree or higher grade AVB	5 (26.3)
Fluoroscopy duration (min)	7 (6, 7)
QRS duration (ms)	
Baseline	139.3 ± 28.0
RVP	176.2 ± 21.4
LBBP	120.9 ± 15.2
LBBP threshold (V at 0.4 ms)	
LBBP threshold at the implantation	0.7 ± 0.3
LBBP threshold during last follow up	0.8 ± 0.2
Devices	
ICD	1 (5.3)
Pacemaker	9 (47.4)
CRT-P	7 (36.8)
CRT-D	2 (10.5)

*Values are mean ±SD or median (IQR). LVEF, left ventricular ejection fraction; RVP, right ventricular pacing; AVB, atrioventricular block; LBBP, left bundle branch pacing; ICD, implantable cardiac defibrillator; CRT-P, cardiac resynchronization therapy-pacing; CRT-D, cardiac resynchronization therapy-defibrillator*.

### ECG Changes After LBBP Upgrade

Eleven patients had an intrinsic rhythm, with a narrow QRS in five, right bundle branch block (RBBB) in three, LBBB in two, and interventricular conduction delay (IVCD) in one (Supplementary Table 1). In patients with an escape rhythm, eight patients had a wide QRSd. Compared with the native QRSd of 139.3 ± 28.0 ms, the mean paced QRSd was wider with RVP (176.2 ± 21.4 ms, *P* < 0.001) and shortened to 120.9 ± 15.2 ms with LBBP (*P* = 0.006). Pre-implant mean QRS axis of all patients was 32.63° (IQR: 0°-70.5°) and was 5.3° by RVP (IQR: −66.00°-80.5°) and kept stable at 63.84° after LBBP (IQR: 41.5°-73°). The percentage of patients with normal QRS axis was 84.2% (*n* = 16) on native and decreased to 26.3% (*n* = 5) on RVP (*p* = 0.0001) and was increased to 78.9% (*n* = 15) on LBBP (*p* = 0.001).

Eleven patients with native conduction had normal R-wave transitions between V3 and V4, while during RVP, 13/19 patients had an R/S transition from V5 to V6. During LBBP, 10 patients were noted to have a normal R/S transition between lead V3 and V4, while nine patients had an R/S transition between leads V1 and V2.

### Echocardiographic Changes After LBBP Upgrade

**Figure 2:**
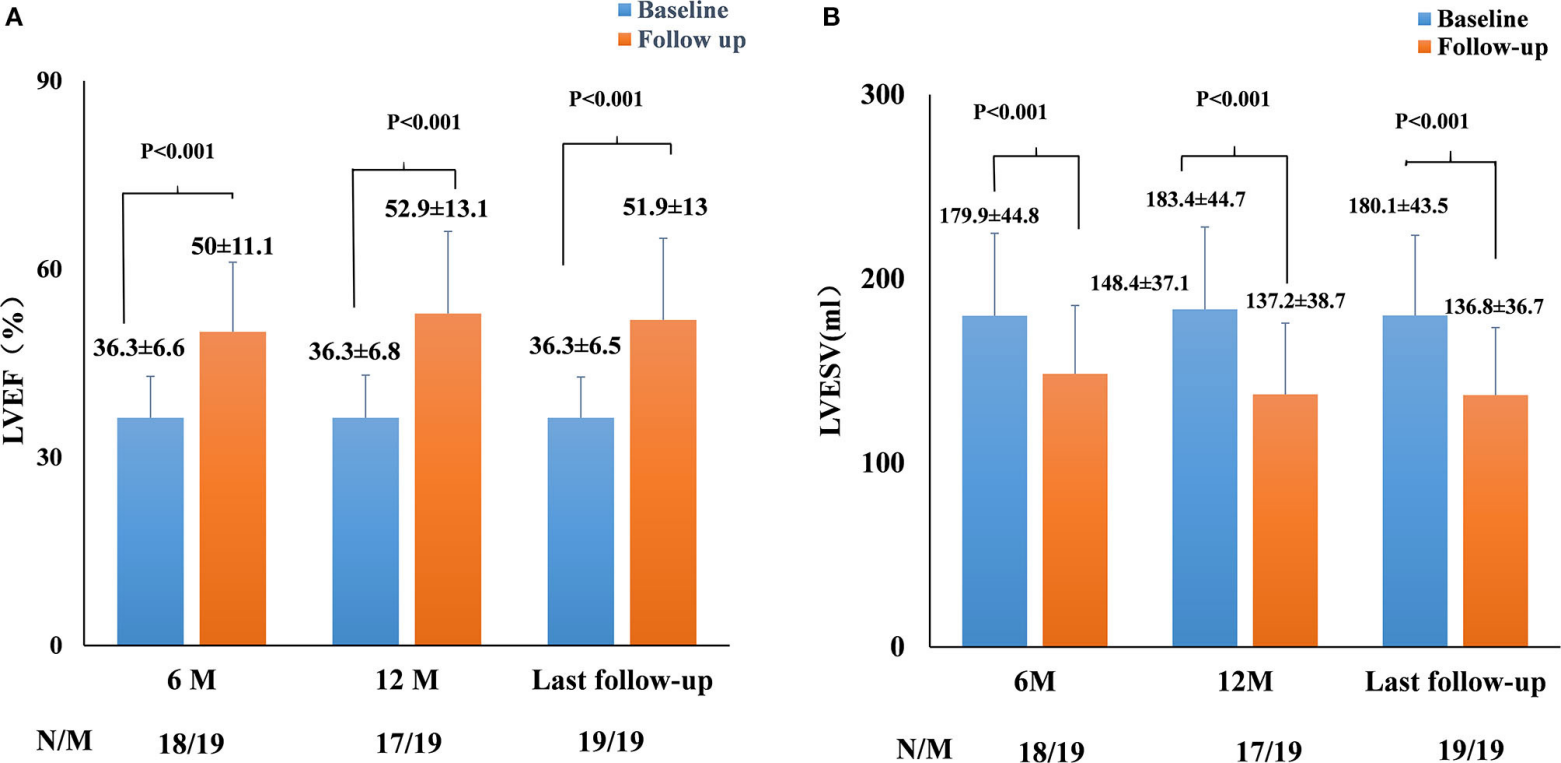
Paired left ventricular ejection fraction (LVEF) **(A)** and left ventricular end-systolic volume (LVESV) **(B)** in all pacing-induced cardiomyopathy (PICM) patients at implant and during the median 12-month follow-up. *N*, the number of patients who had completed the follow-up of 6 and 12 months; *M*, the number of patients who completed the follow-up of 6 and 12 months. Data were available in 18/19 patients at 6-month follow-up, in 17/19 patients at the 12-month follow-up, and in 19/19 patients at the median 12-month follow-up.

**Figure 3:**
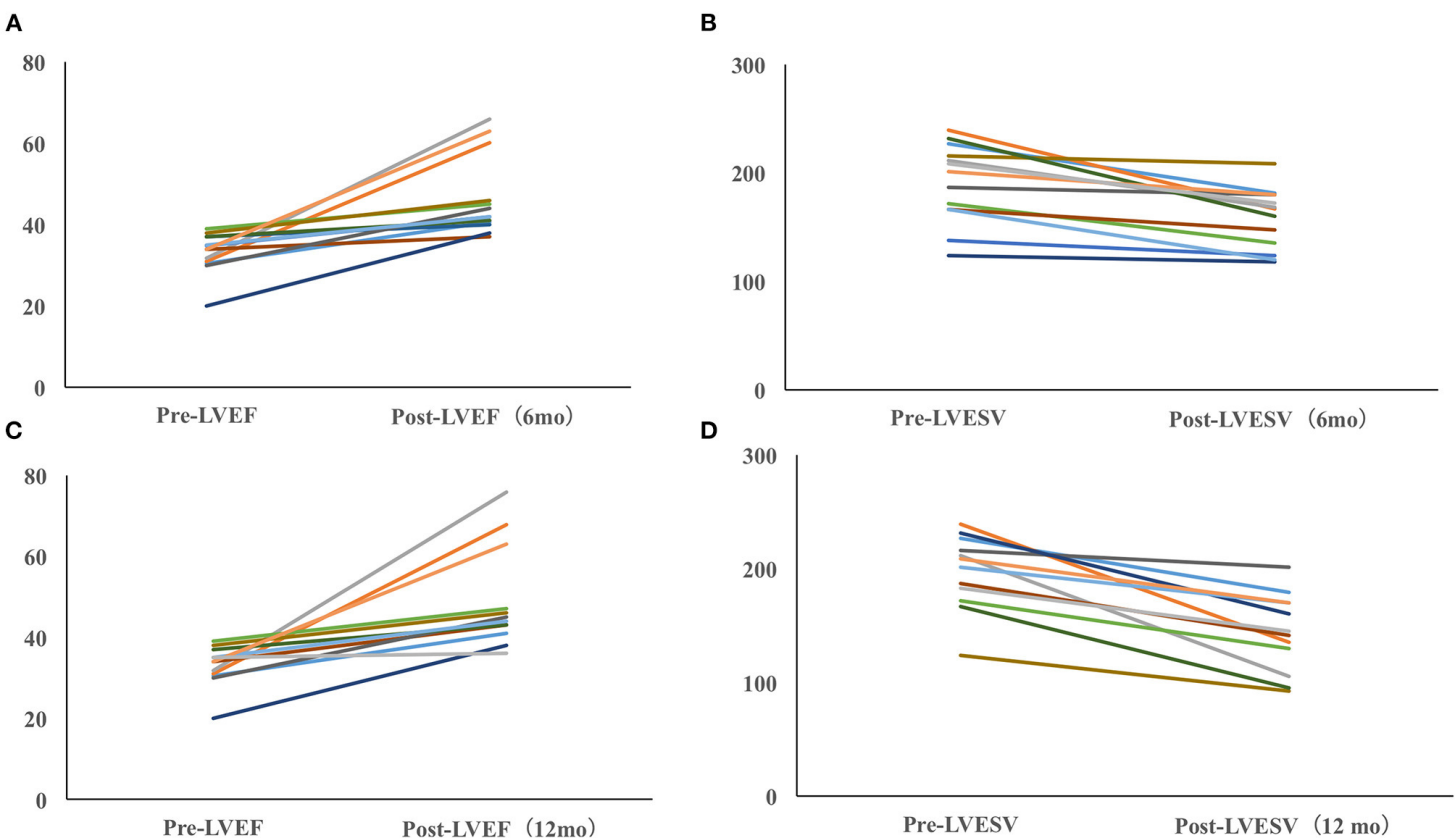
Dynamic echocardiographic changes in PICM patients with baseline LVEF <40%. Improvement of LVEF **(A)** and LVESV **(B)** in PICM patients with LVEF < 40% (6 months). Improvement of LVEF **(C)** and LVESV in PICM patients with LVEF < 40% (12 months) **(D)**. Data were available in 13/14 patients at the 6-month follow-up and in 12/12 patients at the last follow-up.

The echocardiographic measurements are summarized in Figures 2, 3. All patients completed 6-month follow-up, and the mean LVEF was increased from 36.3% ± 6.6% to 50.0% ± 11.1% (*p* < 0.001, *n* = 18) (Figure 2), and the LVESV was reduced from 179.9 ± 44.8 to 148.4 ± 37.1 ml (*p* < 0.001, *n* = 18) (Figure 2). In patients with complete 12-month follow-up, the mean LVEF increased from 36.3% ± 6.8% to 52.9% ± 13.1% (*p* < 0.001, *n* = 17) and the LVESV was reduced from 183.4 ± 44.7 to 137.2 ± 38.7 ml (*p* < 0.001, *n* = 17) (Figure 2). The LVEF increased from 36.3% ± 6.5% to 51.9% ± 13.0% (*P* < 0.001) with LVESV reduced from 180.1 ± 43.5 to 136.8 ± 36.7 ml (*P* < 0.001) during last follow-up, while an improvement in LVEF by ≥5% was observed in 17 patients (89.5%) and a super response was observed in five patients (26.3%).

### Clinical Outcomes and Lead Complications

During a median follow-up of 12 months, NYHA functional class was improved from 2.8 ± 0.6 to 2.1 ± 0.6 (*p* = 0.02). The number of patients with moderate-to-severe HF (NYHA III–IV) decreased from 73.7% (14/19) at baseline to 21.1% at 12 months (2/19) (*P* < 0.001). Of 18 patients who had diuretics before LBBP upgrade, nine patients were noted to have a reduction in diuretic dosage and another seven patients stopped diuretics completely. There was no difference in the number of patients taking angiotensin-converting enzyme inhibitor/angiotensin receptor antagonist (ACEI/ARB) and β-blockers pre- and post-LBBP. In the year preceding LBBP upgrade, five patients (5/19) experienced at least one HFH, and only one patient (1/19) experienced HFH after LBBP (*P* = 0.027). One patient died 12 months after LBBP implantation due to ischemic stroke.

As shown in Table 2, the mean acute LBBP capture threshold, sensed R-wave amplitude, and lead impedance maintained stable. There were no major complications during the implantation or the study period.

**Table 2:** Lead parameters at the baseline and during the follow-up.

**LBBP lead**	**Baseline** **(*N* = 19)**	**6-month** **(*N* = 18)**	**12-month** **(*N* = 17)**
Pacing threshold, V/0.4 ms	0.7 ± 0.3	0.7 ± 0.2	0.8 ± 0.2
Sensing, mV	12.7 ± 4.2	11.8 ± 3.5	11.5 ± 4.2
Impedance, Ohm	625.1 ± 118.8	621.8 ± 94.1	622.8 ± 92.1

## Discussion

The key findings from our study are as follows:

1. LBBP could be performed safely in 95% (19/20) of patients with infranodal AVB who developed PICM.
2. LBB pacing beyond the site of AVB had a low threshold and no loss of left bundle branch capture during the median 12-month follow-up.
3. Favorable echocardiographic indices with improvement in EF, reduction in LV size, and improvement in NYHA class were observed in PICM patients by LBBP.

LBBP was feasible in 95% of patients with infranodal AVB regardless of a narrow or wide QRS escape. LBBP captured the conduction system beyond the site of block, maintained LV synchrony, and reversed electrical dyssynchrony caused by RVP. LBBP improved LV function in PICM patients except for one patient with a native IVCD (Supplementary Table 1).

After LBBP upgrade, 10 patients developed a normal QRS transition and nine patients demonstrated an R/S transition between leads V1 and V2. In our study, a normal QRS axis was maintained with LBBP in most cases and reversed PICM as we previously reported (14). LBBP deliberately targeted the more proximal left bundle in our study, while some LBBP case reports showed left axis deviation for pacing sites located near the left posterior branch (14, 23).

HBP could improve HF symptoms in LBBB as well as AF patients combined with atrial ventricular nodal (AVN) ablation (24, 25). Shan et al. (7) reported on 11 patients with PICM, after the upgrade to permanent HBP, that average LVEF improved and LVEDD decreased. The concerns regarding lead dislodgement, high threshold, and need for lead revisions remained the main problems of HBP in long-term follow-up (11, 17). HBP was feasible acutely in up to 77% of patients with infranodal block (9). Permanent HBP may not be able to pace truly distal to the site of block, particularly in patients with infranodal AVB, and leaves one with the practical concern for progression of distal His-Purkinje conduction disease (9).

LBBP paced beyond the conduction block (12, 15, 26) is an alternative method for delivering ventricular resynchronization comparable to HBP but with stable and lower capture thresholds and a higher success rate (Table 2) (12, 27, 28), while conventional HBP may fail to pace beyond the block site in patients of infranodal AVB (12, 17, 27). In addition, LBBP other than HBP provides more operational space for AVN ablation (29).

In our study, no patients had lead dislodgment, perforation, or threshold increase during a median 12-month follow-up as indicated (14, 30). LBBP is deep enough to penetrate the septum with capture of both the left bundle and the left ventricular septum (22). LVSP provides acute hemodynamic improvement and electrical resynchronization as well as maintains left ventricular function (31).

In a recent case report, LBBP reversed PICM from RVP (15). In our study, PICM patients had a mean drop of 25.9% in LVEF with HF symptoms, and a reduction of LVEF was improved after LBBP upgrade. In our study, all patients completed 6-month follow-up, 89.5% (17/19) patients completed the 12-month follow-up, and the mean follow-up in the study was 13.5 ± 6.2 months, which showed the beneficial effects of LBBP in PICM.

BVP seems to have a beneficial effect on left ventricular reverse remodeling, and the maximal effect of BVP is optimized by maximal fusion from LV lead pacing fusing with the intrinsic right bundle branch in patients with typical LBBB and QRSd longer than 150 ms (32). BVP could not maintain synchrony in patients with a native narrow QRS (6) and leads to non-physiological ventricular activation in AVB patients. Fusion with intrinsic conduction by optimized AV intervals plays an important role in determining the benefit of BVP, which is limited in AF and AVB patients (32), while this could be achieved by HBP or LBBP with narrow QRS or typical LBBB and HF (33, 34). In addition, the role of BVP in patients with AVB and mild-to-moderate impairment in left ventricular function remains controversial, and comparisons of clinical outcomes in such patients with BVP vs. RVP remain mixed (35, 36).

LBBP is a reliable physiological pacing strategy for AVB (13, 14, 26, 27) or HF patients with typical LBBB (16, 28). Maintaining and restoring physiological LV activation is an essential prerequisite in patients with LV dysfunction. Compared with LVSP, LBBP deliverers more physiological LV activation that is comparable to HBP. Moreover, LBBP can be optimized to further improve cardiac synchrony (37). Incorporation and programming of LBBP lead instead of coronary sinus lead into the LV port in a standard cardiac resynchronization therapy-defibrillator (CRT-D) or cardiac resynchronization therapy-pacing (CRT-P) system was feasible for synchronization in PICM patients. We believe that the current data highlights the role for permanent LBBP as a strategy to achieve cardiac synchronization in PICM patients even with infra-Hisian block. Our recent report indicates HBP and LBBP delivered more effective electrical resynchronization compared to BVP in HF patients with LBBB. LBBP was associated with more stable and lower pacing thresholds than those of HBP (28). However, in our study, there was no randomized or direct comparison of biventricular pacing and LBBP.

### Study Limitations

This was a retrospective, multicenter, observational study with limited number of patients. The high success rates of LBBP achieved by experienced operators in our three centers need to be replicated in large pilot studies. Additionally, the success rates and the clinical outcomes of LBBP in this population must be interpreted with caution due to its retrospective study design. A dedicated prospective study is therefore warranted to answer this question. Another limitation of the study was the lack of a direct comparison to BVP as the standard treatment for PICM.

## Conclusions

This retrospective, multicenter, observational study demonstrates the beneficial effect and feasibility of LBBP in PICM patients with infranodal AVB. LBBP paced beyond the site of AV block with a low and stable capture threshold in 12 months as well as a high success rate of implantation in infranodal AVB patients.

## Data Availability Statement

The original contributions presented in the study are included in the article/Supplementary Material, further inquiries can be directed to the corresponding authors.

## Ethics Statement

The studies involving human participants were reviewed and approved by the institutional review board. The patients/participants provided their written informed consent to participate in this study. Written informed consent was obtained from the individual(s) for the publication of any potentially identifiable images or data included in this article.

## Author Contributions

WH, GF, and XC conceived and designed the experiments. YY, SW, YS, XS, JZ, and BW analyzed and interpreted the data. YY, SW, XC, PS, and KE wrote or edited the manuscript. All authors contributed to the article and approved the submitted version.

## Conflict of Interest

The authors declare that the research was conducted in the absence of any commercial or financial relationships that could be construed as a potential conflict of interest.

## Abbreviations

HBP: His bundle pacing
LV: Left ventricular
RV: right ventricular
PICM: pacing-induced cardiomyopathy
AVB: atrioventricular block
LBBP: left bundle branch pacing
QRSd: QRS duration
HF: heart failure
CRT: cardiac resynchronization therapy
BVP: biventricular pacing
LVSP: left ventricular septal pacing
ICD: implantable cardiac defibrillator
RAD: right axis deviation
HFH: heart failure hospitalization
IQR: interquartile range
RBBB: right bundle branch block
LBBB: left branch bundle block
IVCD: interventricular conduction delay.
