# Supplementary material for: Feasibility and Outcomes of Upgrading to Left Bundle Branch Pacing in Patients With Pacing-Induced Cardiomyopathy and Infranodal Atrioventricular Block
Source: Front Cardiovasc Med. 2021 Jun 14;8:674452. doi: 10.3389/fcvm.2021.674452 (PMC8236829; doi:10.3389/fcvm.2021.674452)
Supplement: Supplementary file 1 [file Table_1.DOCX]

**Supplementary Table 1**

| Patient ID | Age | | Gender | Pre  RVP EF% | RVP （%） | Pre-  LBBP EF% | Pre-  LBBP-NYHA | ECG  QRS  pattern | Intrinsic  Or  Escape rhythm | QRSd  Native | QRSd  RVP | QRSd LBBP | HBP  Threshold | Device | LBBP  port | Diuretics  baseline | Follow-up  （Mo） |
| --- | --- | --- | --- | --- | --- | --- | --- | --- | --- | --- | --- | --- | --- | --- | --- | --- | --- |
| EF<40% | |  |  |  |  |  |  |  |  |  |  |  |  |  |  |  |  |
| 1 | | 78 | F | 62.9 | 51 | 30.5 | 3 | RBBB | Intrinsic | 129 | 160 | 135 | 2.5 | CRT-P | LV | YES | 18 |
| 2 | | 64 | F | 62 | 55 | 31 | 4 | Narrow | Intrinsic | 108 | 192 | 121 | 3.0 | CRT-D | LV | YES | 12 |
| 3 | | 70 | M | 55 | 83 | 31.8 | 2 | LBBB | Intrinsic | 174 | 200 | 130 | 3.0 | CRT-P | LV | YES | 12 |
| 4 | | 59 | M | 56.8 | 100 | 34.9 | 3 | Narrow | Intrinsic | 108 | 168 | 121 | 2.5 | PM | RV | YES | 6 |
| 5 | | 71 | M | 59 | 100 | 39 | 3 | Wide | Escape | 124 | 160 | 116 | 3.5 | ICD | A | YES | 12 |
| 6 | | 83 | F | 70.4 | 100 | 37 | 3 | Narrow | Intrinsic | 113 | 142 | 140 | 1.5 | CRT-P | LV | YES | 6 |
| 7 | | 76 | F | 71.7 | 100 | 34 | 3 | Wide | Escape | 132 | 168 | 119 | 2.5 | CRT-P | LV | YES | 12 |
| 8 | | 63 | M | 73 | 97 | 30 | 2 | Wide | Escape | 175 | 180 | 142 | 2.5 | PM | RV | YES | 12 |
| 9 | | 84 | F | 58 | 100 | 35 | 3 | IVCD | Intrinsic | 139 | 147 | 112 | None | PM | RV | YES | 12 |
| 10 | | 76 | F | 61 | 100 | 38 | 2 | Wide | Escape | 154 | 182 | 124 | 2.5 | CRT-P | LV | YES | 12 |
| 11 | | 75 | M | 62 | 100 | 20 | 3 | Wide | Escape | 180 | 200 | 130 | None | CRT-D | A | YES | 12 |
| 12 | | 78 | M | 69 | 90 | 37 | 3 | Narrow | Intrinsic | 98 | 190 | 105 | 2.5 | CRT-P | LV | YES | 12 |
| 13 | | 49 | M | 56 | 100 | 35 | 3 | Wide | Escape | 192 | 222 | 140 | 4.0 | PM | RV | YES | 12 |
| 14 | | 74 | M | 57.1 | 97 | 34 | 2 | RBBB | Intrinsic | 154 | 176 | 121 | 3.5 | CRTP | LV | YES | 12 |
| EF≥40% | |  |  |  |  |  |  |  |  |  |  |  |  |  |  |  |  |
| 15 | | 68 | F | 60 | 100 | 45.3 | 2 | Narrow | Intrinsic | 104 | 146 | 101 | 3.5 | PM | RV | No | 12 |
| 16 | | 66 | M | 62 | 100 | 46.4 | 3 | Wide | Escape | 130 | 184 | 138 | 3.0 | PM | RV | YES | 12 |
| 17 | | 69 | M | 56.3 | 100 | 45 | 4 | LBBB | Intrinsic | 164 | 170 | 105 | 3.5 | PM | RV | YES | 24 |
| 18 | | 64 | M | 55 | 99 | 43 | 3 | RBBB | Intrinsic | 140 | 200 | 112 | 3.0 | PM | RV | YES | 12 |
| 19 | | 67 | F | 74.6 | 100 | 42 | 2 | Wide | Escape | 129 | 160 | 86 | 4.0 | PM | RV | YES | 34 |

EF= Ejection fraction; RVP= Right ventricular pacing; LBBP=Left bundle branch pacing; RBBB=Right bundle branch block; LBBB=Left bundle branch block； IVCD=Intraventricular Conduction Delay; QRSd=QRS duration; PM=Pacemaker; ICD=implantable cardiac defibrillator; CRT-P=Cardiac resynchronization therapy- Pacing; CRT-D= Cardiac resynchronization therapy- Defibrillator; RV=Right ventricle; LV Left ventricle; A=Atrium.
